# Local Observability Analysis of Star Sensor Installation Errors in a SINS/CNS Integration System for Near-Earth Flight Vehicles

**DOI:** 10.3390/s17010167

**Published:** 2017-01-16

**Authors:** Yanqiang Yang, Chunxi Zhang, Jiazhen Lu

**Affiliations:** The Science and Technology on Inertial Laboratory, School of Instrumentation Science and Opto-electronics Engineering, Beijing University of Aeronautics and Astronautics, Beijing 100191, China; yangyq@buaa.edu.cn (Y.Y.); zhangchunxi@buaa.edu.cn (C.Z.)

**Keywords:** star sensor installation errors, local observability analysis, Kalman Filtering, near-earth flight vehicles

## Abstract

Strapdown inertial navigation system/celestial navigation system (SINS/CNS) integrated navigation is a fully autonomous and high precision method, which has been widely used to improve the hitting accuracy and quick reaction capability of near-Earth flight vehicles. The installation errors between SINS and star sensors have been one of the main factors that restrict the actual accuracy of SINS/CNS. In this paper, an integration algorithm based on the star vector observations is derived considering the star sensor installation error. Then, the star sensor installation error is accurately estimated based on Kalman Filtering (KF). Meanwhile, a local observability analysis is performed on the rank of observability matrix obtained via linearization observation equation, and the observable conditions are presented and validated. The number of star vectors should be greater than or equal to 2, and the times of posture adjustment also should be greater than or equal to 2. Simulations indicate that the star sensor installation error could be readily observable based on the maneuvering condition; moreover, the attitude errors of SINS are less than 7 arc-seconds. This analysis method and conclusion are useful in the ballistic trajectory design of near-Earth flight vehicles.

## 1. Introduction

The strapdown inertial navigation system (SINS) has emerged as the most attractive choice for autonomous navigations, particularly for military applications due to its immunity to external interference. However, the inertial instruments errors and initial misalignment errors of SINS lead to accumulated errors over time [1]. Global Positioning System (GPS) is the most popular system used for the integration with SINS to enhance the performance and reliability of the navigation system [2]. In practice, the anti-jamming problems and degraded accuracy in hostile environments make the SINS/GPS integration system a poor choice for autonomous and precise navigation. In comparison to GPS, the celestial navigation system (CNS) is accurate, autonomous, reliable, inexpensive and practically independent of all external inputs, but requires low dynamic or no rotation motion [3]. As a result, the SINS/CNS integrated navigation can take advantage of these methods and achieve high precision in near-Earth flight vehicles [4,5].

Advancement in Optoelectronics and image processing techniques has enabled the development of Charged Coupled Device (CCD) star sensor which can provide the arc-seconds level accuracy of attitude in the inertial frame [6]. The accuracy of a SINS/CNS integration system degrades to the level of arc-minutes without calibration of the installation error angles between SINS and the star sensor [7]. Besides, the installation error calibrated on the ground would undergo changes after the launch of the flight vehicle and large acceleration [8]. The star sensor measurement error induced by its installation error is considerably larger than its own error in the SINS/CNS integration system. The installation error angles, as one of the main factors, degrade the measurement accuracy of the star sensor in the practical application [9]. At the same time, the observability analysis of the installation error is more difficult due to its definition in a body frame, rather than a navigation frame.

Currently, there is not enough literature data to design estimating methods of star sensor installation error, and its observability is rarely compared to other related inertial device errors in SINS/CNS integration for a near-Earth flight vehicle. Gerald and Shuster put forward an algorithm that can make full use of Satellite redundant attitude information to calibrate the star-sensor installation error on an orbit for a satellite. The method applied to satellites is not completely available in an SINS/CNS system for near-Earth flight vehicles [10]. As shown in [11], the misalignments of the star tracker are input as the system states, and the federated filter algorithm is used to estimate the state errors in the SINS/CNS/GNSS (Global Navigation Satellite System) integrated navigation system. An integration approach of SINS/CNS by Uscend Kalman Filter (UKF), Extend Kalman Filter (EKF) and neural network respectively was proposed by Fang [12,13,14], and these methods require large computation efforts when applied in practical cases. Khan Badshah gave a method for fusing the data of SINS and CNS with a modified measurement model based on Linearized Kalman Filter (LKF) without considering the star sensor installation error [15]. Considering the complicated circumstances of near-Earth flight, in order to achieve optimal attitude accuracy, it is necessary to estimate the star sensor installation error, and put forward its observable condition in the case of SINS/CNS. The concept of local observability and locally observable assertion is introduced in [16,17]. For near-Earth flight vehicles, star sensors could be frequently allowed to use shorter measurement intervals [18]. Moreover, a locally observable system is sure to be globally observable, while a globally observable system is not sure to be locally observable [2]. So the observability of this paper stands for local observability. The rank of observability matrix is also used to determine local observability [19]. Consequently, an improved observability analysis of the installation error based on the eigenvalues of the observability matrix for a near-Earth flight vehicle is presented in this study. The simplest measurement schemes of the star sensor for a near-Earth flight vehicle are designed in order to estimate on-line and compensate for the installation error between SINS and a star sensor. Simulations show that the estimated value of errors are in good agreement with the true value, and the platform error angles are less than 7 arc-seconds over the measurement schemes which include rolling and heading adjustment for LFOV. In this work, an integration algorithm for a conventional star sensor is proposed in a local level mechanization. A linearized error model of SINS is used for analysis of the stand-alone SINS and integrated system. KF is used for errors estimation and analysis of the integrated system. The measurement model of KF is developed with the installation error between SINS and the star sensor.

This paper is categorized into five different sections. In Section 2, the integration algorithm is deduced, and Kalman Filtering for the integrated system is designed. The local observability analysis method is described and an observable condition is proposed in detail in Section 3. Simulation results are produced and discussed in Section 4. Finally, the paper is concluded in Section 5.

## 2. The Principles of the SINS/CNS Integration Algorithm

### 2.1. Preview

#### 2.1.1. Reference Coordinate System Definition

The star sensor is able to continuously provide the body attitude as aided navigation information through coordinate transformation with respect to the inertial coordinate system. The coordinate frames used in this paper are defined as follows:

Geocentric inertial frame (i-frame) has its origin at the center of the Earth and is non-rotating with respect to the fixed stars.

Earth frame (e-frame) is a coordinate frame which has its origin at the center of the Earth and has axes which are fixed in the Earth.

Navigation frame (n-frame) is the wander azimuth (WA) frame which is rotated with respect to the local geodetic frame *z* axis by the WA angle.

Computer frame (c-frame) is the local level frame at the computed position.

Platform frame (p-frame) is the navigation frame built by the computed attitude of the SINS.

The body frame (b-frame) is the frame in which the accelerations and angular rates are generated by SINS.

Star sensor frame (s-frame) has its origin at the center of the image plane of the star sensor with *y*-axis normal to the image plane. Its *x* and *y* axes meet the right-hand system.

#### 2.1.2. Definition of Installation Error Angles between SINS and the Star Sensor

$\mu^{b}={\left[\begin{array}{ccc} \mu_{x}^{b} & \mu_{y}^{b} & \mu_{z}^{b} \end{array}\right]}^{T}$ is the installation error vector between SINS and the star sensor in b-frame, and the transition matrix from the s-frame to b-frame can be written as:

(1) $$C_{s}^{b}=\left[\begin{array}{ccc} \cos\mu_{y}^{b}\cos\mu_{z}^{b}-\sin\mu_{y}^{b}\sin\mu_{x}^{b}\sin\mu_{z}^{b} & \cos\mu_{y}^{b}\sin\mu_{z}^{b}+\sin\mu_{y}^{b}\sin\mu_{x}^{b}\cos\mu_{z}^{b} & -\sin\mu_{y}^{b}\cos\mu_{x}^{b}\\ -\cos\mu_{x}^{b}\sin\mu_{z}^{b} & \cos\mu_{x}^{b}\cos\mu_{z}^{b} & \sin\mu_{x}^{b}\\ \sin\mu_{y}^{b}\cos\mu_{z}^{b}+\cos\mu_{y}^{b}\sin\mu_{x}^{b}\sin\mu_{z}^{b} & \sin\mu_{y}^{b}\sin\mu_{z}^{b}-\cos\mu_{y}^{b}\sin\mu_{x}^{b}\cos\mu_{z}^{b} & \cos\mu_{y}^{b}\cos\mu_{x}^{b} \end{array}\right]$$

Using small-angle approximation for $\mu^{b}$ and neglecting the higher order errors result in its simplified form:

(2) $$C_{s}^{b}\approx \left[\begin{array}{ccc} 1 & \mu_{z}^{b} & -\mu_{y}^{b}\\ -\mu_{z}^{b} & 1 & \mu_{x}^{b}\\ \mu_{y}^{b} & -\mu_{x}^{b} & 1 \end{array}\right]=[I-(\mu^{b}\times )]\to (\mu^{b}\times )=\left[\begin{array}{ccc} 0 & -\mu_{z}^{b} & \mu_{y}^{b}\\ \mu_{z}^{b} & 0 & -\mu_{x}^{b}\\ -\mu_{y}^{b} & \mu_{x}^{b} & 0 \end{array}\right]$$

In order to facilitate the analysis in this paper, the direction of installation error angle is defined as the rotation axis shown in subscript.

#### 2.1.3. Definition of Celestial Angles and the Star Vector

**Figure 1:**
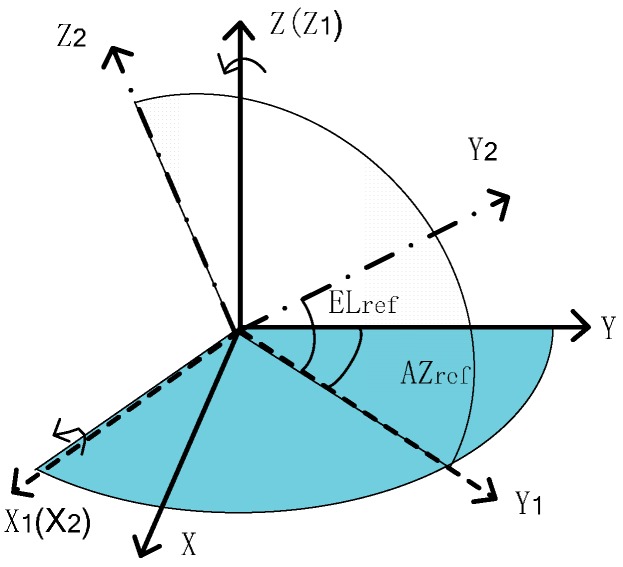
The definition of celestial angles.

The y axis rotates the angle of AZ around the opposite direction of the z axis in reference frame o−xyz, building an intermediate coordinate system $o-x_{1}y_{1}z_{1}$. The $y_{1}$ axis rotates the angle of EL around the $x_{1}$ axis in frame $o-x_{1}y_{1}z_{1}$, forming a new frame $o-x_{2}y_{2}z_{2}$ as shown in Figure 1. The axis of $y_{2}$ should be the axis of starlight. The star vector in o−xyz can be written as:

(3) $$l_{ref}=\left[\begin{array}{c} \cos EL_{ref}\sin AZ_{ref}\\ \cos EL_{ref}\cos AZ_{ref}\\ \sin EL_{ref} \end{array}\right]$$

where $EL_{ref}$ and $AZ_{ref}$ are called the celestial angles in the reference frame.

### 2.2. Principle of the SINS/CNS Integration Algorithm

Generally, the vector of starlight $l_{s}$ in the s-frame can be obtained autonomously by the star sensor. Thus, the vector of starlight $l_{p}$ in the p-frame can be described as follows [15],

(4) $$l_{p}={\hat{C}}_{b}^{n}l_{s}=[I-(\psi \times )][I+(\mu^{n}\times )]l_{c}$$

where $\mu^{n}$ is the installation error between SINS and the star sensor in the n-frame. $\mu^{n}\times$ is the skew symmetric matrix of $\mu^{n}$. ψ is the platform misalignment angle of SINS. ψ× is the skew symmetric matrix of ψ. ${\hat{C}}_{b}^{n}$ is the attitude matrix determined by SINS. $C_{b}^{n}$ is the true value of the attitude matrix. The vector of starlight $l_{c}$ in the c-frame can be obtained by the position resolved from SINS, the star catalog and the almanac tabulate [15].

The corresponding measurement information of celestial angles is the main objective of this integration algorithm. It can be written as

(5) $$\Delta EL=EL_{p}-EL_{c},\Delta AZ=AZ_{p}-AZ_{c}$$

Substituting Equations (3) and (5) into Equation (4), using small-angle approximation for ΔEL and ΔAZ and neglecting the higher order errors gives the result in its simplified form as follows in reference [15]

(6) $$z=\left[\begin{array}{c} \Delta EL\\ \Delta AZ \end{array}\right]=\left[\begin{array}{ccc} -\cos AZ_{c} & \sin AZ_{c} & 0\\ -\tan EL_{c}\sin AZ_{c} & -\tan EL_{c}\cos AZ_{c} & 1 \end{array}\right]\cdot \left[\begin{array}{cc} I_{3*3} & -T \end{array}\right]\cdot \left[\begin{array}{c} \psi_{x}\\ \psi_{y}\\ \psi_{z}\\ \mu_{x}^{b}\\ \mu_{y}^{b}\\ \mu_{z}^{b} \end{array}\right]$$

where T is the transition matrix $C_{b}^{n}$ from the b-frame to the n-frame. z will be considered as the basic measurement for the rest of the paper.

### 2.3. Design of Kalman Filtering with Installation Error between SINS and CNS

A Kalman Filtering, assuming a small error perturbation in the SINS dynamic system, is used for estimation and correction of the system errors. Considering a discrete-time process governed by the linear stochastic equation, the linear measurement model is as follows,

(7) $$X_{k+1}=\Phi_{k}X_{k}+w_{k}$$

where $X_{k}={\left[\begin{array}{cccccccc} \Delta \theta & \Delta h & \Delta v & \psi & \varepsilon^{b} & gSF^{b} & gMA^{b} & \mu^{b} \end{array}\right]}^{T}$ is the state vector. $\Phi_{k}$ is the discrete transition matrix of the state vector derived from the linearized error equation of SINS in the n-frame. $w_{k}$ is the white noise in the state transition process. In the above formulation, Δθ,Δh,Δv,ψ are position, altitude, velocity and attitude error vectors, respectively; $\varepsilon^{b},gSF^{b},gMA^{b}$ are gyro biases, scale factor and misalignments in b-frame, respectively; $\mu^{b}$ is the installation error vector between SINS and star sensor in b-frame.

The SINS/CNS Kalman Filtering is used to estimate the platform misalignment error ‘**ψ**’ of SINS in terms of the star vector observation. The measurement formula is as follows:

(8) $$z_{k}={\left[\Delta EL\;\Delta AZ\right]}^{T}=\left[\begin{array}{cccc} 0_{2\times 6} & A & 0_{2\times 6} & -A \end{array}\cdot T\right]\cdot X_{k}+v_{k}$$

where

$$A=\left[\begin{array}{ccc} -\cos AZ_{c} & \sin AZ_{c} & 0\\ -\sin EL_{c}\sin AZ_{c} & -\sin EL_{c}\cos AZ_{c} & \cos EL_{c} \end{array}\right]$$

$$z_{k}=\left[\begin{array}{c} EL_{p}-EL_{c}\\ cos(EL_{c})[AZ_{p}-AZ_{c}] \end{array}\right]$$

$v_{k}$ is the white noise in the measurement process. To keep the paper reasonably concise, other equations of Kalman Filtering are presented in reference [15].

## 3. Local Observability Analysis of the Installation Errors

Formally, a system is said to be locally observable, for any possible sequence of state vectors, if the current state can be determined in finite time only by the measurements. It can be evaluated by forming the system observability matrix and checking the rank of the matrix [16]. A full rank observability matrix means that all the states can be estimated when the noise characteristic of the system is known. It is noticeable that the observability of the star sensor installation errors is only related to the platform's error angles. Therefore, we can choose the installation errors and platform's error angles as the states. The star sensor and the near-Earth flight vehicle undergo strap down installation. The vehicle cannot perform pose adjustment when star sensor conducts measurement for the accuracy of star sensor. Hence, we can suppose that platform's error angles and installation errors remain constant in the ultra-short time period for simplicity, i.e.,:

(9) $$\begin{array}{l} \dot{\psi }=0\\ \dot{\mu }=0 \end{array}$$

It also should be noted that local observability is related to the number of stars or the field of view. Accordingly, two categories are included in the analysis below. One is a large field of view (LFOV) star sensor which measures two or more star vectors and the other is a narrow field of view (NFOV) star sensor measuring only one star vector.

### 3.1. Local Observability of LOFV Star Sensor

According to the principle of SINS/CNS, the following equations can be obtained in the three different-time measurements of two star vectors guaranteeing that the rank of observability matrix is equal to 6. It is determined by the process of measuring a star. Proof is given below with properties of a matrix rank and orthogonal matrix.

(10) $$\left[\begin{array}{c} z_{1}\\ z_{2}\\ z_{3} \end{array}\right]=\left[\begin{array}{c} A_{1}\cdot \left[I_{3\times 3}-T(t_{1})\right]\\ A_{2}\cdot \left[I_{3\times 3}-T(t_{2})\right]\\ A_{3}\cdot \left[I_{3\times 3}-T(t_{3})\right] \end{array}\right]\cdot \left[\begin{array}{c} \psi_{3\times 1}\\ \mu_{3\times 1} \end{array}\right]$$

The observability matrix of an equation can be expressed as follows,

(11) $$O_{obs}=A\cdot B=\left[\begin{array}{ccc} A_{1} & 0_{4\times 3} & 0_{4\times 3}\\ 0_{4\times 3} & A_{2} & 0_{4\times 3}\\ 0_{4\times 3} & 0_{4\times 3} & A_{3} \end{array}\right]\cdot \left[\begin{array}{c} B_{1}\\ B_{2}\\ B_{3} \end{array}\right]$$

where

$$\begin{array}{l} A_{i}={\left[\begin{array}{ccc} -\cos AZ_{ci} & \sin AZ_{ci} & 0\\ -\sin EL_{ci}\sin AZ_{ci} & -\sin EL_{ci}\cos AZ_{ci} & \cos EL_{ci}\\ -\cos A{Z^{\prime }}_{ci} & \sin A{Z^{\prime }}_{ci} & 0\\ -\sin E{L^{\prime }}_{ci}\sin A{Z^{\prime }}_{ci} & -\sin E{L^{\prime }}_{ci}\cos A{Z^{\prime }}_{ci} & \cos E{L^{\prime }}_{ci} \end{array}\right]}_{4\times 3}\\ B_{i}={\left[I_{3\times 3}-T(t_{i})\right]}_{3\times 6}(i=1,2,3) \end{array}$$

The rank of the observability matrix meets the following inequality Equation (12).

(12) $$\{\begin{array}{c} \mathrm{Rank}(O_{obs})=\mathrm{Rank}(\mathrm{AB})\leq \min\text{\{}\mathrm{Rank}(A),\mathrm{Rank}(B)\}\\ \mathrm{Rank}(O_{obs})=\mathrm{Rank}(\mathrm{AB})\geq \mathrm{Rank}(A)+\mathrm{Rank}(B)-n,(n=9) \end{array}$$

Apparently, the rank of **A** is 9, and the rank of **B** is less than or equal to 6. Therefore, the rank of the observation matrix is only determined by the rank of **B**.

(13) $$\mathrm{Rank}(O_{obs})=\mathrm{Rank}(B)$$

Row elementary operations on **B** matrix yield:

(14) $$B={\left[\begin{array}{c} \begin{array}{c} I_{3\times 3}-T(t_{1})\\ I_{3\times 3}-T(t_{2}) \end{array}\\ I_{3\times 3}-T(t_{3}) \end{array}\right]}_{9\times 6}\Rightarrow {\left[\begin{array}{cc} I_{3\times 3} & -T(t_{1})\\ 0 & T(t_{1})-T(t_{2})\\ 0 & T(t_{1})-T(t_{3}) \end{array}\right]}_{9\times 6}$$

Based on the reduced order theorem of the matrix rank, the rank of **B** can be given below

(15) $$\mathrm{Rank}(B)=\mathrm{Rank}(I_{3\times 3})+\mathrm{Rank}\left(\left[\begin{array}{c} T(t_{1})-T(t_{2})\\ T(t_{1})-T(t_{3}) \end{array}\right]\right)$$

We rewrite the above formula with the theorem of the block matrix rank, then we have:

(16) $$\mathrm{Rank}(B)=3+\mathrm{Rank}\left(\left[\begin{array}{c} T(t_{1})-T(t_{2})\\ T(t_{1})-T(t_{3}) \end{array}\right]\right)\leq 3+\mathrm{Rank}\left(T(t_{1})-T(t_{2})\right)+\mathrm{Rank}\left(T(t_{1})-T(t_{3})\right)$$

If T is an attitude matrix and if it is orthogonal, then the difference can be expressed as:

(17) $$T(t_{1})-T(t_{2})=T(t_{1})(I-T{\left(t_{1}\right)}^{T}T(t_{2}))$$

Note that the attitude matrix is the direction cosine matrix and its rank is equal to 3. Hence, the rank of the difference of attitude matrices is presented as follows

(18) $$\mathrm{Rank}\left(T(t_{1})-T(t_{2})\right)=\mathrm{Rank}((I-T{\left(t_{1}\right)}^{T}T(t_{2}))$$

The matrix $T{\left(t_{1}\right)}^{T}T(t_{2})$ of the above formula is an order 3 real orthogonal matrix the determinant of which is equal to 1, and the number of eigenvalues is 1 or 3. The rank of $T(t_{1})-T(t_{2})$ can be calculated after diagonalization of the matrix $T{\left(t_{1}\right)}^{T}T(t_{2})$,

(19) $$\mathrm{Rank}\left(T(t_{1})-T(t_{2})\right)=\mathrm{Rank}((I-T{\left(t_{1}\right)}^{T}T(t_{2}))=\left\{\begin{array}{c} 0,\;eig(T{\left(t_{1}\right)}^{T}T(t_{2}))=\left\{1,1,1\right\}\\ 2,eig(T{\left(t_{1}\right)}^{T}T(t_{2}))=\left\{1,\lambda^{*},\lambda \right\},(s.t.\lambda^{*}\lambda =1) \end{array}\right\}$$

where *eig* is the function of matrix eigenvalue. $\lambda^{*}$ and λ are mutual complex conjugates . The results are summarized by the following conclusion in the observation of two star vectors.

Case LOFV-1: The rank of the observation matrix is 3, when the attitude matrix remains constant in three different time measurements. Accordingly, not all of the installation errors can be observable. The observability is mainly constrained by the rank of the **B** matrix. The lack of posture adjustment during three different time measurements determines that the rank of the B matrix is equal to 3.

(20) $$\begin{array}{l} \mathrm{Rank}(O_{obs})=\mathrm{Rank}(B)=3\\ s.t.T(t_{1})=T(t_{2})=T(t_{3}) \end{array}$$

Case LOFV-2: One of the installation error angles is not observable, in which case just one of the attitude angles changes during three different time measurements. The one which is unable to be estimated is predictable. Obviously, the direction of the unobservable installation error angle is parallel to the rotation of the virtual axis of the star sensor.

(21) $$\begin{array}{l} \mathrm{Rank}(O_{obs})=\mathrm{Rank}(B)=5\\ s.t.T(t_{3})=C_{r}(\alpha_{2})\cdot T(t_{2})=C_{r}(\alpha_{1})\cdot T(t_{1}),\alpha_{1}\neq \alpha_{2} \end{array}$$

$C_{r}(\alpha )$ is defined as the transition matrix that rotates at the angle of α around the *r* axis.

Case LOFV-3: Two of the attitude angles change during the three different time measurements, all of the installation errors are clearly observable.

(22) $$\begin{array}{l} \mathrm{Rank}(O_{obs})=\mathrm{Rank}(B)=6\\ s.t.T(t_{3})=C_{r2}(\alpha_{2})\cdot T(t_{2})=C_{r1}(\alpha_{1})\cdot T(t_{1}),r2\neq r1 \end{array}$$

This conclusion gives a sufficient and necessary condition to confirm the installation errors’ observability for the LFOV star sensor in a SINS/CNS integrated system. The above results are suitable for more than 2 star vectors. The increasing number of star vectors does not change observability, but can improve the degree of observability and the capacity of the resisting disturbance.

#### 3.2. The Observability of the NFOV Star Sensor

The observability matrix of Equation (11) can be obtained for the three different time measurements of one star vector, and it can be expressed as follows,

(23) $$O_{obs}=A\cdot B=\left[\begin{array}{ccc} A_{1} & 0_{2\times 3} & 0_{2\times 3}\\ 0_{2\times 3} & A_{2} & 0_{2\times 3}\\ 0_{2\times 3} & 0_{2\times 3} & A_{3} \end{array}\right]\cdot \left[\begin{array}{c} B_{1}\\ B_{2}\\ B_{3} \end{array}\right]$$

where

$$\begin{array}{l} A_{i}={\left[\begin{array}{ccc} -\cos AZ_{ci} & \sin AZ_{ci} & 0\\ -\sin EL_{ci}\sin AZ_{ci} & -\sin EL_{ci}\cos AZ_{ci} & \cos EL_{ci} \end{array}\right]}_{2\times 3}\\ B_{i}={\left[I_{3\times 3}-T(t_{i})\right]}_{3\times 6}(i=1,2,3) \end{array}$$

The rank of observability matrix satisfies the following inequalities.

(24) $$\{\begin{array}{c} \mathrm{Rank}(O_{obs})=\mathrm{Rank}(\mathrm{AB})\leq \min\text{\{}\mathrm{Rank}(A),\mathrm{Rank}(B)\}\\ \mathrm{Rank}(O_{obs})=\mathrm{Rank}(\mathrm{AB})\geq \mathrm{Rank}(A)+\mathrm{Rank}(B)-n,(n=9) \end{array}$$

Apparently, the rank of **A** is 6, and the rank of **B** is less than or equal to 6. Therefore, the rank of the observation matrix is still determined by the rank of **B**.

(25) $$\mathrm{Rank}(B)-3\leq \mathrm{Rank}(O_{obs})\leq \mathrm{Rank}(B)$$

The results are summarized by the following conclusion.

Case NOFV-1: When the attitude matrix remains constant during three different time measurements, none of the installation errors are observable.

(26) $$\begin{array}{l} 0\leq \mathrm{Rank}(O_{obs})\leq \mathrm{Rank}(B)=3+\mathrm{Rank}\left(\left[\begin{array}{c} T(t_{1})-T(t_{2})\\ T(t_{1})-T(t_{3}) \end{array}\right]\right)=3\\ s.t.T(t_{1})=T(t_{2})=T(t_{3}) \end{array}$$

Case NOFV-2: At least one of the installation error angles are not observable, in which case one of the attitude angles changes during the three different time measurements.

(27) $$\begin{array}{l} 2\leq \mathrm{Rank}(O_{obs})\leq \mathrm{Rank}(B)=3+\mathrm{Rank}\left(\left[\begin{array}{c} T(t_{1})-T(t_{2})\\ T(t_{1})-T(t_{3}) \end{array}\right]\right)=5,\\ s.t.T(t_{3})=C_{r}(\alpha_{2})\cdot T(t_{2})=C_{r}(\alpha_{1})\cdot T(t_{1}),\alpha_{1}\neq \alpha_{2} \end{array}$$

Case NOFV-3: Two of the attitude angles change during three different time measurements, all of the installation errors between SINS and CNS may be clearly observable.

(28) $$\begin{array}{l} 3\leq \mathrm{Rank}(O_{obs})\leq \mathrm{Rank}(B)=3+\mathrm{Rank}\left(\left[\begin{array}{c} T(t_{1})-T(t_{2})\\ T(t_{1})-T(t_{3}) \end{array}\right]\right)=6\\ s.t.T(t_{3})=C_{r2}(\alpha_{2})\cdot T(t_{2})=C_{r1}(\alpha_{1})\cdot T(t_{1}),r2\neq r1 \end{array}$$

The above-mentioned conclusion gives a necessary condition observable for the NFOV star sensor installation error in a SINS/CNS integrated system. The installation error around the optical axis is not observable in any case for NFOV.

## 4. Simulation and Discussion

In this section, a simulation is carried out to check the observability analysis of the installation errors. In order to make the simulation result more credible, most of the error terms are included in a simulation error source. The simulating condition is set as follows:

A Fiber Optic Gyroscope (FOG) is chosen as a test gyro, the constant and random drifts of which are 0.03°/h, 0.006°/h, respectively. The scale factor error is 20 ppm, and the installation error is 5 arc-seconds. The constant and random biases of accelerometers are 50 μg and 15 μg. The scale factor error is 50 ppm, and the installation error is 5 arc-seconds. The SINS measurements are generated with a sample rate of 100 Hz. The precision of the star sensor is 5 arc-seconds. NFOV is about 2° and LFOV is about 20°. The error of the star catalog is 1arc-seconds. The misalignment error between INS and star sensor is about 200 arc-seconds. The star sensor measurement update is provided at a frequency of 10 Hz. The initial position error is set as 100 m. The initial attitude error is 1 arc-minute. One measurement operation lasts 5 s.

**Figure 2:**
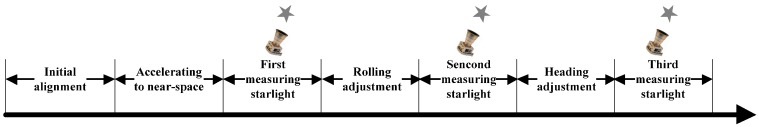
Simulation procedure.

According to typical maneuver of near-Earth flight vehicles, the movement track of a vehicle in the simulation is designed as Figure 2, ground initial alignment for 260 s and accelerating to near-Earth space. The vehicle then moved with a horizontal velocity that changed with small discontinuous accelerating and decelerating vertical velocity and pose adjustment. We measured the star vector three times until the end of the experiment. Simulations were performed in three cases based on the Monte Carlo method. Case 1 represents the situation that the attitude matrix remains constant during three different time measurements. Case 2 represents the situation that the roll attitude angles changes after the first measurements and the two remaining measurements are performed with the same attitude. Case 3 represents the situation that the roll attitude angles changes after the first measurements and the heading attitude angles changes after the second measurements. The estimated values of the installation errors are illustrated in Table 1, Table 2 and Table 3 respectively, which indicate the observability in the SINS/CNS.

In Case 1, the near-Earth flight vehicle just finished the first starlight measurement. Three installation errors of the star sensor would not be observed. The observability is unable to be improved whether for LFOV or NFOV. The simulation results shown in Table 1 are in agreement with the analysis and observability is mainly influenced by posture adjustment times instead of the increasing number of stars.

In Case 2, the near-Earth vehicle had two different observation attitudes, the heading of which changed. Compared with Case 1, the observability is improved greatly. Two installation error angles of the star sensor can be observed for the LFOV star sensor. The unobservable error $\mu_{z}^{b}$ is just parallel to heading axis. So the optimal observability can be based on more than two star vectors. However, only one of the installation error angles can be estimated due to $\mu_{y}^{b}$ being parallel to the optical axis for NFOV. Thus, the simulation results shown in Table 2 are in agreement with the analysis and it is still mainly influenced by the viewing posture for LFOV.

In Case 3, the near-Earth vehicle had three different observation attitudes, the heading and rolling of which are changed. The results are shown in Table 3. Compared with Case 1 and Case 2, the observability is improved. All the installation error angles can be observed for the LFOV star sensor due to the times of posture adjustment being equal to 2. It must be noted, however, that the installation error is still not fully observable owing to the unobservable installation error being parallel to the optical axis in the case of NFOV. Consequently, a simple position adjustment around the optical axis can make the observability optimal for NFOV. With more than one star vector, the installation errors are completely observable. In summary, the number of star vectors should be greater than or equal to 2, and the times of posture should be greater than or equal to 2.

**Figure 3:**
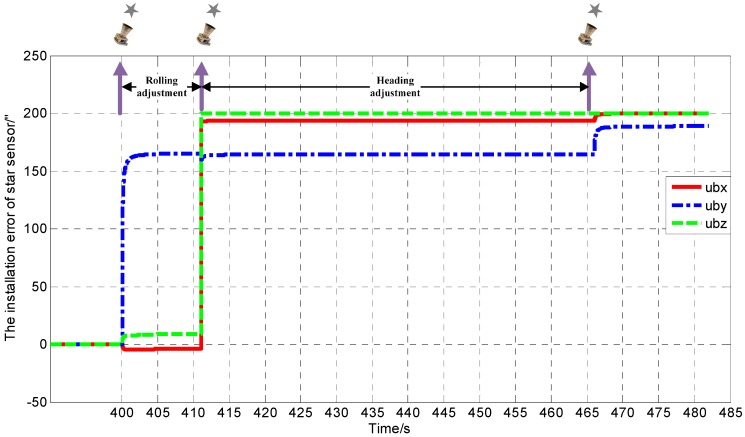
The installation error convergence curve for Case 3.

Simulation results verify the correctness of observability analysis above. The installation error convergence curve is given in Figure 3 measuring the starlight three times for LFOV. At the end of the first rolling posture adjustment, the estimated value of installation error $\mu_{y}^{b}$ parallel to the rolling axis is unchanged, the same as $\mu_{z}^{b}$ parallel to the heading axis at the end of second heading posture adjustment. Other installation error angles basically converged to the true value. At the end of the second heading posture adjustment, the estimated installation error $\mu_{y}^{b}$ is close to its true value, and still a little weaker than other installation error angles parallel to the optical axis. In all cases, the installation error is observable by means of rolling adjustment and heading adjustment for LFOV.

The previous conclusion showed a remarkable efficiency in the estimation of the installation errors. In order to verify that the accuracy of SINS/CNS can be clearly increased, it is necessary to undertake simulations under three conditions, namely: SINS, SINS/CNS integration method without considering star sensor installation errors (SINS/CNS-1), SINS/CNS integration method considering star sensor installation errors (SINS/CNS-2).

**Figure 4:**
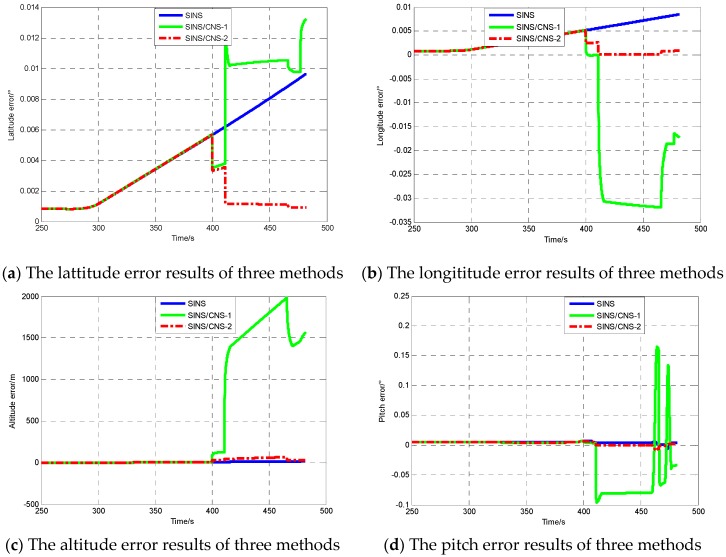

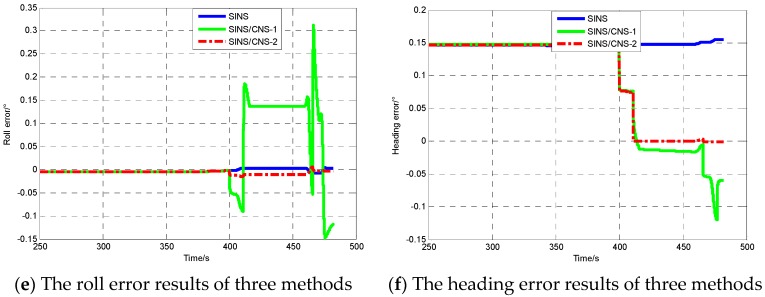
Comparision of the navigation errors between strapdown inertial navigation system (SINS), SINS/CNS integration method without considering star sensor installation errors (SINS/CNS-1), SINS/CNS integration method considering star sensor installation errors (SINS/CNS-2).

The simulation results are only intended to emphasize the fact that it is essential to precisely estimate the installation error for the SINS/CNS integration system. In this specific case, it is not only a filtering algorithm issue, and previous cited work could help only in part. The real need is for a better modeling, i.e., for the proposed algorithm that includes installation errors. As can be seen in Figure 4d–f, attitude errors and azimuth error are completely estimated along with vehicle maneuvering in the case of SINS/CNS-2, increasing the observability of star sensor installation errors in order to improve the accuracy of the CNS/SINS integrated system. According the principle of SINS/CNS integration, observability of the velocity and position errors is poor. From the simulation results in Figure 4a–c, position accuracy was also improved significantly with increasing attitude accuracy. The main reason for this is that the velocity as well as the position errors due to platform misalignment are compensated for. However, the slight increase in longitude error owing to the accelerometer cannot be compensated for after the third calibration measurement in the case of SINS/CNS-2. For the case of SINS/CNS-1, the integration method is not convergent as a result of inaccurate measurement, and navigation accuracy is inferior to the case of SINS. It is essential to precisely estimate the installation errors for the SINS/CNS integration system.

The detailed navigation error results of simulations are illustrated in Table 4. As in the case of SINS/CNS-1, the heading error is close to the installation errors of the star sensor, which may restrict the actual accuracy of SINS/CNS. The position error is greater than in the case of SINS due to inaccurate estimation of inertial device errors caused by the star sensor installation errors. Compared with SINS, the SINS/CNS-2 integration method presented in this work can achieve the same precision as the star sensor. The attitude errors are accurately compensated for and reduced from 559.48 arc-seconds to 2.18 arc-seconds. The position errors are limited to less than 103 m in about a 220-s flight. Consequently, the result is indicative of effective estimation about the star sensor installation errors. Meanwhile, the CNS/SINS integration method and observability conclusion are validated.

## 5. Conclusions

In this paper, local observability analysis of star sensor installation error is performed on the rank of observability matrix. Compared to numerical simulation methods, the proposed local observability approach can provide much more analytic information. The necessary condition to confirm the installation errors observability is also proposed for the NFOV star sensor. The sufficient and necessary condition to confirm the installation errors’ observability of the implemented Kalman Filtering is demonstrated for the LFOV star sensor. The observability analysis of the installation errors indicates that the installation errors are entirely observable if the times of posture adjustment are more than twice those of the LFOV star sensor. The number of star vectors should be greater than or equal to 2. The unobservable error can be predicted by angular motion analysis. To verify the theoretical results, a simplified SINS/CNS integration algorithm and star sensor measurement process are designed and simulated with the installation error between SINS and the star sensor. The results demonstrate effectiveness for estimation of the installation error. This conclusion can be an effective guidance for flight vehicle path planning. Such an algorithm is required to meet high precision navigation. The strict demonstration of the necessary condition for the local observability of the SINS/CNS integration algorithm provides a more convenient way to predict local observability than numerical analysis of an observable matrix along a certain trajectory. This method can also be used for attitude determination in a satellite.

## Figures and Tables

**Table 1:** The estimated value of installation errors in Case 1 (unit: arc-seconds).

	NFOV	LFOV	
Symbol	One Star Vector	Two Star Vectors	Three Star Vectors
$\mu_{x}^{b}$	189	176.2	176.7
$\mu_{y}^{b}$	2	171.3	174.1
$\mu_{z}^{b}$	138	141.9	142.2

**Table 2:** The estimated value of installation errors in Case 2 (unit: arc-seconds).

	NFOV	LFOV	
Symbol	One Star Vector	Two Star Vectors	Three Star Vectors
$\mu_{x}^{b}$	193	199.1	199.5
$\mu_{y}^{b}$	0.4	194.8	196.9
$\mu_{z}^{b}$	132	146.2	147.4

**Table 3:** The estimated value of installation errors in Case 3 (unit: arc-seconds).

	NFOV	LFOV	
Symbol	One Star Vector	Two Star Vectors	Three Star Vectors
$\mu_{x}^{b}$	197.9	199.8	199.9
$\mu_{y}^{b}$	0.3	194.9	196.8
$\mu_{z}^{b}$	197.3	199.8	199.8

**Table 4:** The near-Earth vehicle navigation errors at the end of flight (unit: arc-seconds).

Error	SINS	SINS/CNS-1	SINS/CNS-2
Latitude/m	1072.24	1469.27	103.49
Longtitude/m	944.00	−1915.69	94.82
Altitude/m	16.72	1564.85	26.48
Pitch/”	14.33	−121.69	4.74
Roll/”	12.01	−424.68	−6.88
Heading/”	559.48	−215.97	−2.18
